# Investigating the Antibacterial Characteristics of Japanese Bamboo

**DOI:** 10.3390/antibiotics11050569

**Published:** 2022-04-24

**Authors:** Raviduth Ramful, Thefye P. M. Sunthar, Kaeko Kamei, Giuseppe Pezzotti

**Affiliations:** 1 Graduate School of Science and Technology, Kyoto Institute of Technology (KIT), Kyoto 606-8585, Japan; 2 Mechanical and Production Engineering Department, Faculty of Engineering, University of Mauritius, Reduit 80837, Mauritius; 3 Ceramic Physics Laboratory, Kyoto Institute of Technology, Kyoto 606-8585, Japan; d0871502@edu.kit.ac.jp (T.P.M.S.); pezzotti@kit.ac.jp (G.P.); 4 Department of Immunology, Graduate School of Medical Science, Kyoto Prefectural University of Medicine, Kyoto 602-0841, Japan; 5 Department of Biomolecular Engineering, Kyoto Institute of Technology, Kyoto 606-8585, Japan; kame@kit.ac.jp; 6 The Center for Advanced Medical Engineering and Informatics, Osaka University, Osaka 565-0871, Japan; 7 Department of Dental Medicine, Graduate School of Medical Science, Kyoto Prefectural University of Medicine, Kyoto 602-8566, Japan; 8 Department of Orthopedic Surgery, Tokyo Medical University, Tokyo 105-8461, Japan

**Keywords:** bamboo, antibacterial, extraction, thermally modified, lignin, FTIR spectroscopy

## Abstract

Natural materials, such as bamboo, is able to withstand the rough conditions posed by its environment, such as resistance to degradation by microorganisms, due to notable antibacterial characteristics. The methods of extraction exert a significant influence on the effectiveness of bamboo-derived antibacterial agents. In this study, the antibacterial characteristics of various types of Japanese bamboo, namely, Kyoto-Moso, Kyushu-Moso and Kyushu-Madake were investigated by considering an extraction and a non-extraction method. The characterization of the efficacy of antibacterial agents of various bamboo samples derived from both methods of extractions was conducted using an in vitro cultured bacteria technique consisting of *E. coli* and *S. aureus*. Antibacterial test results based on colony-forming units showed that antibacterial agents derived from the non-extraction method yielded better efficacy when tested against *E. coli* and *S. aureus*. Most specimens displayed maximum antibacterial efficacy following a 48-h period. The antibacterial agents derived from thermally modified bamboo powder via the non-extraction method showed improved antibacterial activity against *S. aureus* specifically. In contrast, absorbance results indicated that antibacterial agents derived from the extraction method yielded poor efficacy when tested against both *E. coli* and *S. aureus*. From FTIR analysis, characteristic bands assigned to the C-O and C-H functional groups in lignin were recognized as responsible for the antibacterial trait observed in both natural and thermally modified Japanese bamboo powder. Techniques to exploit the antibacterial characteristics present in bamboo by identification of antibacterial source and adoption of adequate methods of extraction are key steps in taking advantage of this attribute in numerous applications involving bamboo-derived products such as laminates and textile fabrics.

## 1. Introduction

Bamboo has a relatively short life span ranging between one to three years under storage conditions as it is affected by both non-living physical and chemical elements as well as living organisms in the ecosystem [1]. Despite its susceptibility to natural degradation, one of the reasons that bamboo grows rapidly and unblemished in nature is its antibacterial properties. This natural immunity against bacteria, especially on its outermost surface, renders bamboo resistant to varieties of bacteria and other biotic agents such as insects, fungi and marine borers in its natural environment.

Given the recognizable anti-bacterial resistance characteristics of natural bamboo, its self-defence against abiotic and biotic factors could be an advantageous feature if fully exploited in various bamboo-derived products. Recently, this antibacterial trait has been used in bamboo-derived products such as in hot-pressed bamboo particle-boards for improved resistance to microorganisms [2,3]. In hot-pressed bamboo particle-board, the antibacterial trait was enhanced by introducing bamboo vinegar into the material. The antibacterial feature is assumed to emerge from the major components present in bamboo vinegar, which is derived from the pyrolysis of bamboo charcoal, such as acetic acid, phenolic compounds and alcohol compounds, among others [2]. Hot-pressed magnetic reconstituted bamboo board, which is produced by applying iron oxide to carbonized bamboo bundle units, was found to display improved mildew resistance and be more suitable for outdoor and other extreme environments compared to simply heat treated boards [3]. Xi et al. observed that natural bamboo fibers with higher hygroscopicity tend to exhibit lower antibacterial performance [4].

Antimicrobial derivatives which originate from plants, such as lignin, are considered safer alternatives than metal-based antimicrobial agents and polymer-based antimicrobial agents given their negligible impact on the environment, wide availability and cost-effectiveness [5,6]. Several studies to determine the antibacterial property of bamboo have identified lignin as being the primary source of the antibacterial compound [1,7,8]. In a research study about the origin of the antibacterial property of bamboo, Afrin et al. identified lignin as the primary source of the antibacterial compound. From FTIR results, the antibacterial property was assumed to stem from the aromatic and phenolic functional groups in lignin [1]. Additionally, given their water insoluble nature, the antibacterial agents are believed to reside in lignin, which is also insoluble in water [7]. In addition to extraction method, previous studies have demonstrated that the antimicrobial potency of lignin is also determined by the plant source [9,10]. A higher yield of monolignols during lignin extraction was found to be essential to maximize antimicrobial characteristics of lignin. The phenolic components present in hydroxyphenyl (H), guaiacyl (G) and syringyl monolignols in lignin are considered to have antimicrobial characteristics [11]. Recent research on the antimicrobial characteristics of lignin found that it was effective on pathogenic microorganisms such as *Escherichia coli* and *Listeria monocytogenes* [12,13].

Numerous investigations on extraction methods to capitalize on the antibacterial trait of lignin have been conducted and remain a field of interest for many researchers to date. Similar methods used in the extraction of lignin involved ethanol fractionation, acid precipitation, and nanoparticle modification, among others [7,14,15,16]. The antibacterial performance of lignin is found to predominantly depend on extraction methods, as reported in several comparative studies [2,7,9,11]. Fractionation through the Kraft method, lignosulphonate, and soda pulping are a few currently used processes by which lignin is chemically extracted [11]. Wang et al. improved the antibacterial activity of bamboo-derived lignin against both Gram-positive and Gram-negative bacteria by using a simple one-step fractionation process [2]. To stimulate the high stability and extended longevity of antibacterial activity in lignin, advanced modifications methods to produce functional hybrid materials are being explored [15,17]. Despite the existence of several proven methods of lignin extraction and purification, the full potential to tap its antimicrobial characteristics in new markets is very sparse and limited.

Naturally, bamboo has recognizable anti-bacterial resistance which can be further investigated in bamboo-derived products for specific applications in which this feature can be advantageous. The aim of this research was to further explore the use of lignin in bamboo, and its associated extraction method to enhance the inherent anti-bacterial resistance characteristics of bamboo. The investigation was principally focused on the antibacterial resistance properties of natural and treated bamboo powder obtained from the Kyoto and Kyushu regions of Japan. The outcome of this study could have direct benefits based on the antibacterial trait of bamboo as a useful feature during the use of raw and recycled bamboo products, in both natural and processed states, for advanced and reconstituted materials.

## 2. Materials and Methods

### 2.1. Sample Preparation

**Figure 1:**
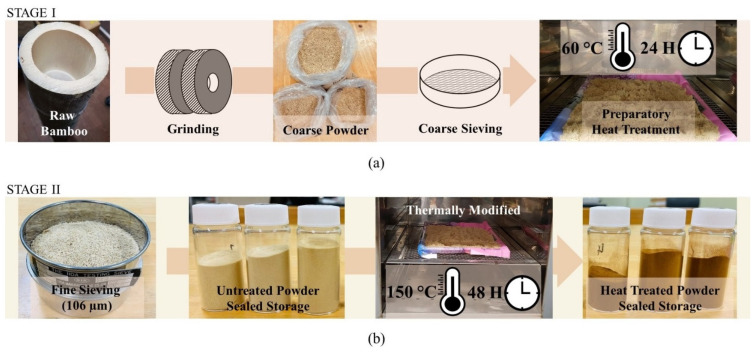
Schematic illustration of methodology of specimen preparation in this study. (**a**) Stage I of the treatment involving grinding, coarse sieving and preparatory heat treatment, and (**b**) Stage II of the treatment involving fine sieving, thermal treatment and sealed storage.

In this investigation, the samples of bamboo used were in powder form derived from ground bamboo culm taken from the two most popular bamboo species in Japan. These were Moso bamboo (*Phyllostachys edulis*) from the Kyoto and Kyushu regions of Japan, and Madake bamboo (*Phyllostachys bambusoides*) from the Kyushu region of Japan. Figure 1 shows the various stages of sample preparation. In first stage, shown in in Figure 1a, the unsieved ground bamboo powder was subjected to a preparatory heat treatment of 60 °C for 24-h to remove the remaining moisture content in the sample.

In the second stage, as illustrated in Figure 1b, the dried samples were subjected to a thorough sieving process with a filter size of 106 µm to uniformize the size of grain particles. One batch of samples was subjected to a heat treatment prior to the antibacterial experiment. The samples were subjected to a heat treatment of 150 °C for a 48-h duration. All prepared specimens were carefully labelled and stored in sealed containers away from sunlight prior to use in the antibacterial test.

### 2.2. Antibacterial Experiment

To investigate the impact of extraction method on the antibacterial efficiency of bamboo, two types of antibacterial agents of bamboo were considered: one derived through the extraction method and one in the natural state.

#### 2.2.1. Extraction Method

In the extraction method, 20 g of fine bamboo powder, mixed with 100 mL of ethanol at 99.95% for 5 days, was filtered to produce a supernatant solution. The supernatant solution was subjected to a rotary evaporator ethanol extraction method to produce the dissolved crude bamboo particles consisting of antimicrobial molecules.

#### 2.2.2. Non-Extraction Method

In this method, 0.02 g of fine bamboo powder was used to test the antibacterial efficiency of bamboo. A similar method was used in the past to test the antibacterial efficiency of bamboo as outlined by Afrin et al. [1] and Xi et al. [4].

### 2.3. Sample Characterization

In this procedure, the same procedures utilized during in vitro bacteria culture and in its pre-characterization stages, were adopted for both extraction and non-extraction methods. However, different verification methods were adopted to check the antibacterial efficacy after extraction and non-extraction methods.

#### 2.3.1. In Vitro Bacteria Culture

Sample characterization for antibacterial efficacy was conducted using the in vitro cultured bacteria technique. In vitro bacteria cultures using two species of bacteria, namely *Staphylococcus aureus* (NBRC 13276) and *Escherichia coli* (E1 NBRC 3972), which are Gram-positive and Gram-negative bacteria, respectively, were cultured using the streak plate method. The bacteria used in this study were cultured in a lab environment. The bacteria were cultured from bacteria beads installed in agar-containing plates followed by an incubation period of 24 h under 37 °C. After 24 h, the colony was collected and mixed with 5 mL of Luria Broth (LB Broth, Sigma-Aldrich, Tokyo, Japan) followed by transfer to a shaking incubator (Southwest Science, Hamilton, NJ, USA) set at 37 °C and 175 rpm for a 24-h period. The second plate was prepared by four sector quadrant streaks using one colony from the first plate. Finally, 100 uL of the bacteria solution were pipetted and mix with 5 mL of sterilized LB Broth medium, and the dilution steps were repeated until an absorbance of 0.3 optical density (OD) was achieved.

#### 2.3.2. Pre-Characterization of Bacterial Culture

**Figure 2:**
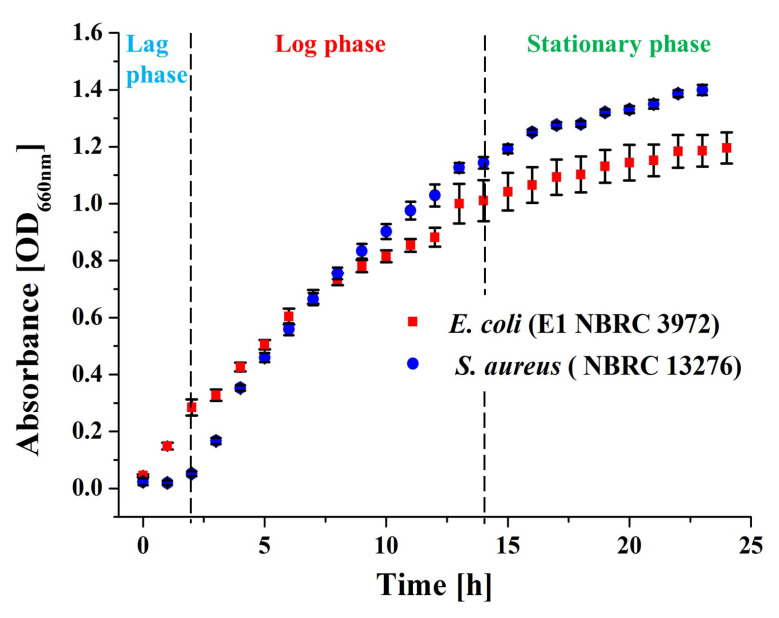
Microbial growth curves of *E. coli* and *S. aureus* indicating the three main periods of microbial activity, namely, the lag phase, log phase and stationary phase, based on optical density values collected by the miniphoto 518R photometer.

In this study, optical density at a wavelength of 660 nm (OD_660nm_) was used to estimate the concentration of bacteria during various growth stages of bacterial culture, the lag phase, log phase and stationary phase, by measuring OD_660nm_ using a photometer. The growth curves of the bacteria were analysed by measuring the optical density (OD) value using miniphoto 518R photometer (TAITEC CORPORATION, Tokyo, Japan). The OD values were recorded at one hour of intervals between 0 and 24 h. Bacteria culture characterization was assessed in terms of a bacterial growth curve, as shown in the Figure 2 below, by plotting the absorbance OD values against time.

From the measured microbial growth count, the data in Figure 2 provide a clear indication of the microbial activity based on the lag, log and stationary phases.

#### 2.3.3. Antibacterial Efficacy—Microbial Growth Count Method (Biological Characterization—Colony Forming Units) Preparation

Sample characterization in terms of antibacterial efficacy was conducted by using two methods. In the extraction method, the samples were diluted starting from 1000 μg to 62.5 μg by microdilution. Four different concentrations were prepared starting from 1000, 500, 250, 125, and 62.5 μg by diluting 500 μL of each concentration with 500 μL of 1% DMSO solution. Then, 100 μL of each concentration was place in a 96 well plate and 100 μL of bacteria solution was added into each well. Two types of bacterial solution, namely *E. coli* and *S. aureus,* were used in this method.

The sample’s optical density (OD) was measured using a microplate reader (Hitachi Ltd., Tokyo, Japan) at 0, 6, 18, 24 and 48 h time intervals. Each sample was replicated thrice to obtain an average reading. The absorbance results of two concentrations, namely C1 and C2 corresponding to 62.5 μg and 1000 μg, respectively, were used for further analysis. The OD data is available online as Supplementary Material File S1. The abbreviations for various types of antibacterial agents of bamboo, assigned based on their location, species and concentration, are shown in Table 1.

In the non-extraction method, finely sieved-bamboo powder in both natural and heat-treated conditions were used. Abbreviations of various types of antibacterial agents of bamboo assigned based on their location, species and treatment modification used in this method are displayed in Table 2.

For each specimen, 0.02 g was added into 2 mL Eppendorf tubes followed by 100 μL of LB Broth and 100 μL of *E. coli* and *S. aureus* bacteria solution. The samples were then run on an Eppendorf shaker at a speed of 1000 rpm and 37 °C for 24 and 48 h. Then the samples were centrifuged using a High-Speed Refrigerated Centrifuge, CR-GIII (Hitachi Ltd., Tokyo, Japan) at 4 °C and 10,000 rpm for 10 min to make sure the powder had settled and the supernatant could be used for analysis. A 100 μL sample of the supernatant was diluted to 10^−5^ by serial dilution and 100 μL of the solution was added to an LB agar plate, spread evenly and incubated at 37 °C for 24 h. The number of colonies was then calculated. Colony-forming units, CFU/mL were calculated using the formula:

CFU/mL = (no. of colonies × dilution factor) × 10

### 2.4. Fourier Transformed Infrared Spectroscopy (FTIR)

To determine the effects of change of chemical constituents of bamboo on its antibacterial efficacy following treatment modification, Fourier transform infrared spectroscopy (FTIR) was conducted using an ATR-FTIR, FTIR-4700, and ATR PRO ONE fitted with a diamond prism (Jasco Co., Tokyo, Japan) with a resolution of 4 cm^−1^ and with 100 scans. Origin (Origin 8.5, OriginLab Co., Northampton, MA, USA) and LabSpec (LabSpec Version 5.58.25 Horiba/Jobin-Yvon, Kyoto, Japan) software were used for spectral analysis and pre-processing of raw data.

## 3. Experimental Results

### 3.1. Extraction Method

**Figure 3:**
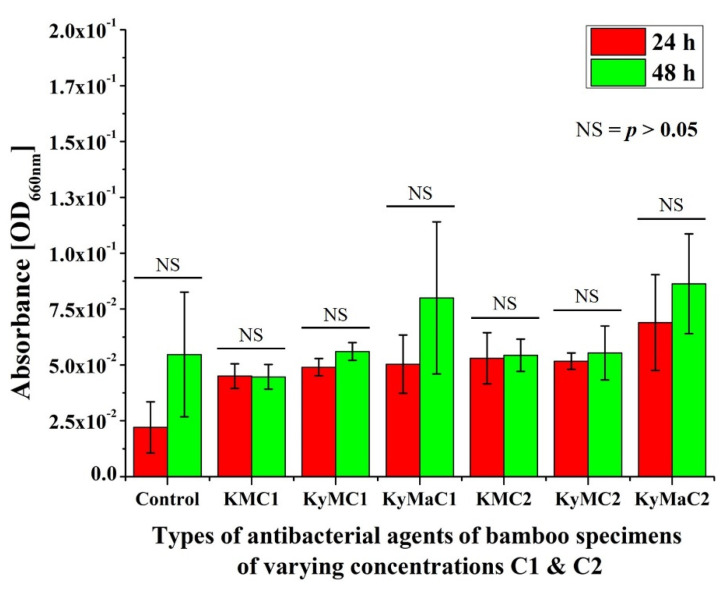
Absorbance results of various types of antibacterial agents of bamboo specimens of differing concentrations, C1 and C2, after in vitro testing with *E. coli* for a 24- and 48-h periods (NS = no significant difference).

The absorbance results of various types of antibacterial agents of bamboo specimens of differing concentrations, C1 and C2, after in vitro testing with *E. coli* for 24 h and 48 h periods are shown in Figure 3. No statistically significant differences in absorbance results could be discerned between the 48 h and 24 h periods with each type of antibacterial agent of bamboo specimens.

**Figure 4:**
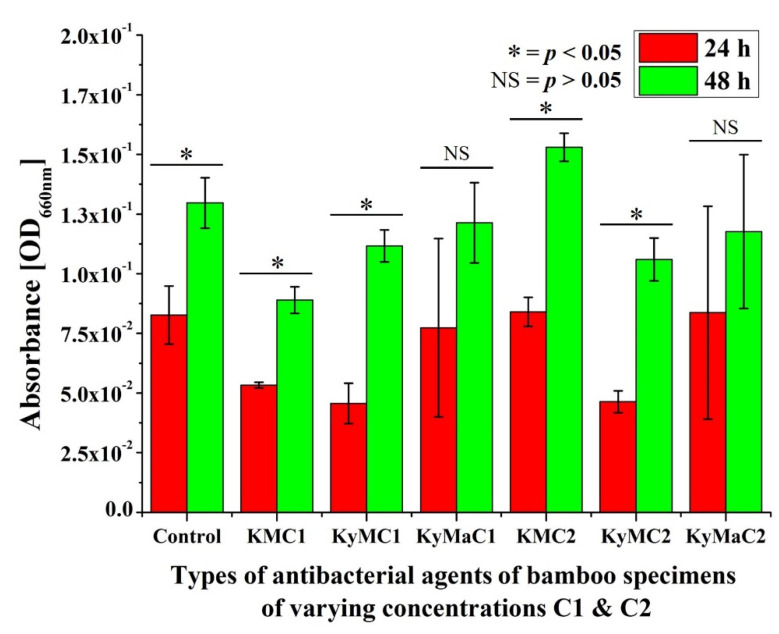
Absorbance results of various types of antibacterial agents of bamboo specimens of differing concentrations, C1 and C2, after in vitro testing with *S. aureus* for 24 h and 48 h periods (* statistically significant difference, NS = no significant difference).

Figure 4 shows the absorbance results of various types of antibacterial agents of bamboo specimens of differing concentrations, C1 and C2, after in vitro testing with *S. aureus* for a 24 h and 48 h periods. In reference to the absorbance results at the 24 h mark, a statistically significant increase in absorbance values was observed after 48 h in control specimens and in Kyoto-Moso and Kyushu-Moso at both concentrations C1 and C2.

### 3.2. Non-Extraction Method

**Figure 5:**
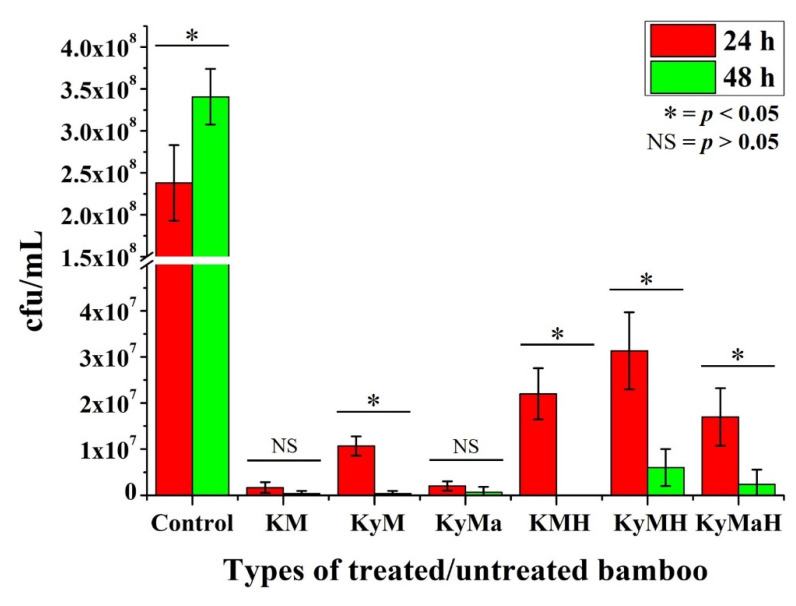
Colony-forming units (CFU) of various types of antibacterial agents of bamboo specimens of varying treatment modifications after in vitro testing with *E. coli* for a 24 h and 48 h periods (* statistically significant difference, NS = no significant difference).

Figure 5 shows the CFU counting results for the bamboo specimens tested against *E. coli.* With reference to the control specimens, all six types of bamboo specimens showed excellent effectiveness against *E. coli* bacteria. In the first 24 h, both natural Kyoto-Moso and Kyushu-Madake bamboo showed higher effectiveness compared to the rest. Hence no significant statistical difference was discerned in these types of specimens between the 24 h and 48 h periods.

Most specimens showed maximum antibacterial characteristics after a 48 h period except heat-treated Kyushu-Madake and Kyushu-Moso specimens. Statistically significant differences in antibacterial activity were noted between the 24 h and 48 h period in the Kyushu-Madake specimen and in heat-treated Kyoto-Moso, Kyushu-Moso and Kyushu-Madake specimens.

**Figure 6:**
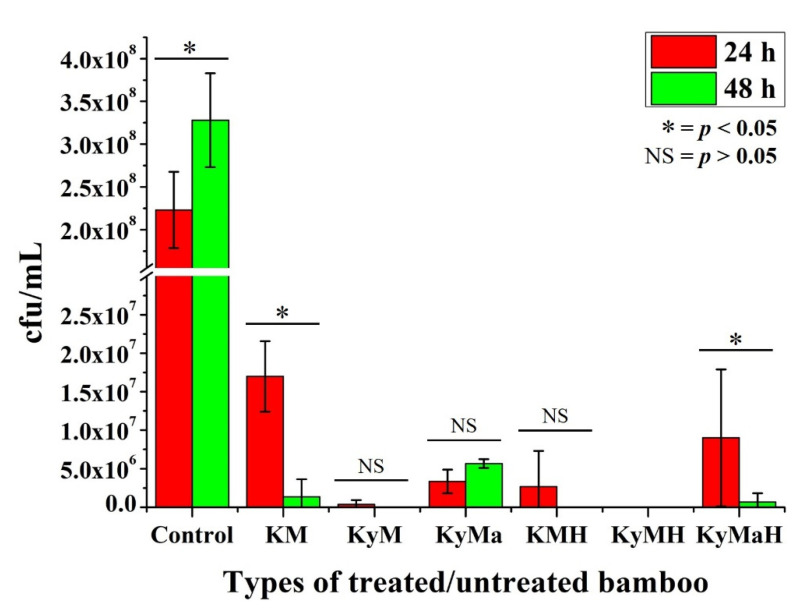
Colony-forming units (CFU) of various types of antibacterial agents of bamboo specimens of varying treatment modifications after in vitro testing with *S. aureus* for 24 h and 48 h periods (* statistically significant difference, NS = no significant difference).

Figure 6 shows the CFU counting results for the bamboo specimens tested against *S. aureus.* With reference to the control specimens, all six types of bamboo specimens showed excellent effectiveness against *S. aureus* bacteria. In the first 24 h, both natural and heat-treated Kyushu-Moso bamboo showed maximum effectiveness compared to the rest. Hence, no significant statistical difference was discerned between natural and heat-treated specimens between the 24 h and 48 h periods.

Most specimens showed maximum antibacterial characteristics after a 48 h period except natural Kyoto-Moso and Kyushu-Madake specimens. Statistically significant differences in antibacterial activity were noted between the 24 h and 48 h periods in natural Kyoto-Moso and heat-treated Kyushu-Madake specimens.

### 3.3. FTIR Analysis

**Figure 7:**
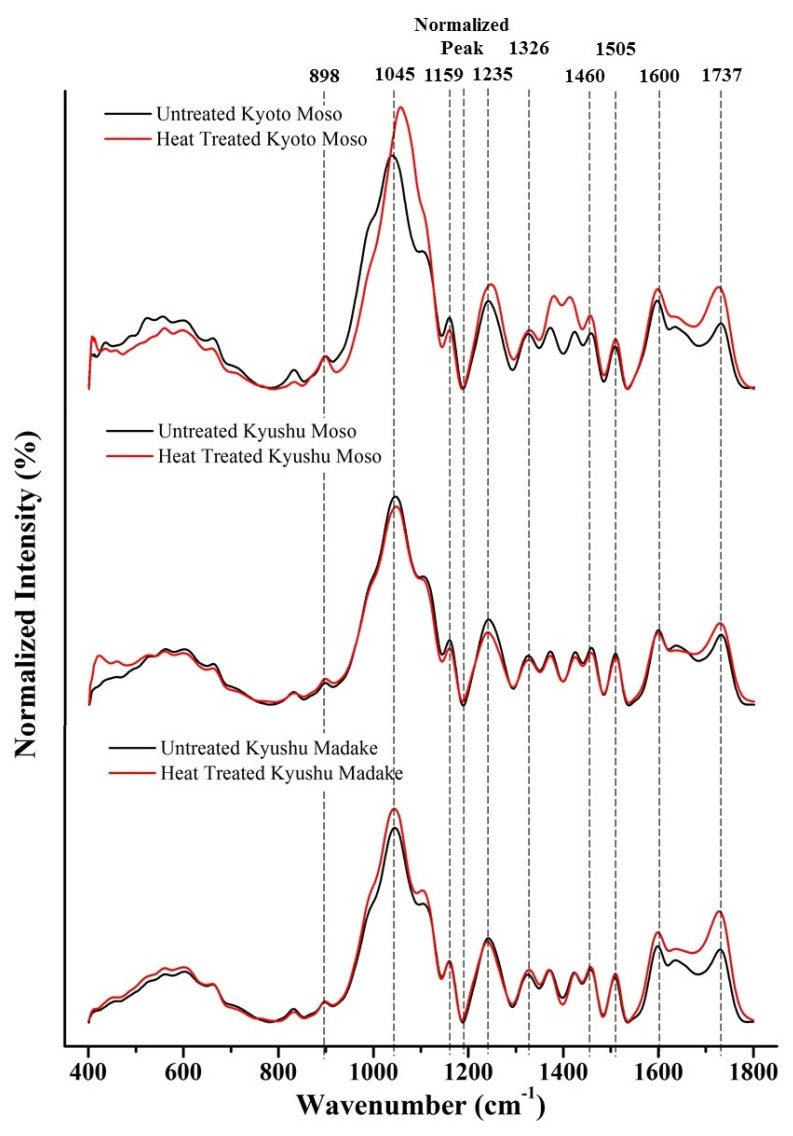
FTIR spectra in the range 400–1800 cm^−1^ of three different types of untreated and thermally modified bamboo specimens, namely, Kyoto Moso, Kyushu Moso and Kyushu Madake bamboo.

Figure 7 shows FTIR spectra in the range of 400–1800 cm^−1^ of three different types of untreated and thermally modified bamboo specimens, namely, Kyoto Moso, Kyushu Moso and Kyushu Madake bamboo.

From the FTIR results in Figure 7, untreated and thermally modified bamboo powder displayed similar molecular footprints to those observed in the FTIR analyses of previous studies involving solid samples [17,18,19]. The characteristic bands of FTIR spectra of the various bamboo specimens in the frequency interval of 400 to 1800 cm^−1^ are shown in Table 3.

Notable changes in the characteristic bands of FTIR spectra at peaks 1045 cm^−1^ and 1737 cm^−1^ were observed following modification by thermal treatment. At the 1045 cm^−1^ peak, which was assigned to C-O and C-H primary alcohol group in guaiacyl lignin, a marked increase was observed in heat-treated Kyoto Moso and Kyushu Madake bamboo [21]. An increase was also observed at peak 1737 cm^−1^ corresponding to C=O carbonyl groups in lignin in all of the three different types of thermally modified bamboo specimens [24].

## 4. Discussion

The colony-forming unit (CFU) counting results of various types of antibacterial agents of treated bamboo specimens after in vitro testing with *S. aureus* for a 24-h and 48 h periods, as shown in Figure 6, was found to be in conformity with the FTIR results shown in Figure 7. The noticeable decrease in the CFU counting results originating from the antibacterial agents of heat-treated Kyoto Moso and Kyushu Madake bamboo is in accordance with the marked increase in lignin observed at peak 1045 cm^−1^ in both Kyoto Moso and Kyushu Madake bamboo following heat treatment. The peak at 1045 cm^−1^ specifically corresponds to C-O and C-H primary alcohol groups in guaiacyl lignin [21]. On the other hand, *E. coli* was found to be less effective against the antibacterial agents of heat-treated bamboo, as observed in Figure 5.

Even though having a lower effectiveness, a marked decrease in the CFU counting results originating from the antibacterial agents of heat-treated Kyoto Moso and Kyushu Madake bamboo was observed compared to Kyushu Moso bamboo, as indicated in Figure 6. This result is in accordance with the previous observation made in the FTIR results of Figure 7 concerning the notable increase in lignin observed at peak 1045 cm^−1^ in both Kyoto Moso and Kyushu Madake bamboo following heat treatment. Based on the above findings, it may be concluded that heat treatment modification does have an influence on the antibacterial performance of bamboo powder. The C-O and C-H functional groups, corresponding to the primary alcohol group in guaiacyl lignin, can thus be considered as the chemical constituent responsible for the antibacterial efficacy in bamboo. This assertion corresponds to observations made in previous studies about the origin of the antibacterial property of bamboo [1], which was postulated to be located in lignin.

### 4.1. Antibacterial Mechanism in Bamboo

In terms of antibacterial mechanism, the sugar content of lignin is assumed to promote and increase its adhesion to the bacterial membrane [28]. From previous studies, the peptidoglycan layer of bacterial cell walls was also able to interact with the glucan content and polysaccharide of the sugar molecules [29]. The presence of various compounds in the structure of lignin, namely phenolic compounds, carboxylic acid containing OH-group and methoxyl and epoxy functional groups containing oxygen, were found to be key factors that influenced the antibacterial property of bamboo [28]. In addition, the effect of thermal modification is assumed to have yielded greater amounts of phenolic carboxylic acids and lignin containing fragments [30]. This was further evidenced through FTIR results in which an increase at peak 1737 cm^−1^, corresponding to a high content of phenolic carboxylic acid OH, was discerned [24,25,26,27,30].

### 4.2. Future Recommendations

Based on the influence of thermal modification on the antibacterial performance of bamboo powder, two approaches to verify and ascertain this observation are proposed. First, to assess the extent of the influence of thermal modification on the antibacterial performance of bamboo powder, further investigation based on varying intensity of treatment modification may be considered. Second, to improve the efficacy of bamboo-derived antibacterial agents, their compatibility with other catalytic reagents may be further explored.

## 5. Conclusions

As a natural material that grows unblemished in nature, bamboo has notable antibacterial traits that can be beneficial in numerous applications if carefully exploited. Extraction methods to exploit the antibacterial agents present in bamboo were found to exert a significant influence on their effectiveness. In this study, an extraction method and a non-extraction method were considered to investigate the antibacterial nature intrinsic to various types of Japanese bamboo, namely Kyoto-Moso, Kyushu-Moso and Kyushu-Madake. In both methods, the antibacterial agents were tested against two species of bacteria, namely *E. coli* and *S. aureus*. From the antibacterial test results, antibacterial agents derived from the non-extraction method yielded better efficacy when tested against *E. coli* and *S. aureus* compared to the antibacterial agents derived from the extraction method. Kyoto-Moso and Kyushu-Madake bamboo were found to be most effective against *E. coli*, while both natural and heat-treated Kyushu-Moso were found to be most efficient when tested against *S. aureus.*

Furthermore, antibacterial agents derived from thermally modified bamboo powder, showed improved antibacterial activity against *S. aureus* specifically. As evidenced by FTIR analysis, and in accordance with previous literature, the characteristic bands at peak 1045 cm^−1^, which have been attributed to lignin, were identified as accountable for the antibacterial trait in both natural & thermally modified bamboo powder. The C-O and C-H functional groups corresponding to the primary alcohol group in guaiacyl lignin were considered the chemical constituents responsible for the antibacterial efficacy in bamboo. The results of this study can be useful in taking advantage of the antibacterial characteristics present in bamboo when applied in numerous practical applications involving bamboo derived products such as laminates and fabrics.

## Figures and Tables

**Table 1:** Abbreviations of various types of antibacterial agents of bamboo based on their location, species and concentration.

	Types of Antibacterial Agents of Bamboo Specimens of Varying Concentrations C1 & C2		
Location	Kyoto	Kyushu	Kyushu
Species	Moso (*Phyllostachys edulis*)	Moso (*Phyllostachys edulis*)	Madake (*Phyllostachys bambusoides*)
Concentration/μg			
62.5	KMC1	KyMC1	KyMaC1
1000	KMC2	KyMC2	KyMaC2

**Table 2:** Abbreviations of various types of antibacterial agents of bamboo assigned based on their location, species and treatment modification.

	Types of Antibacterial Agents of Bamboo Specimens Subjected to Treatment Modification		
Location	Kyoto	Kyushu	Kyushu
Species	Moso (*Phyllostachys edulis*)	Moso (*Phyllostachys edulis*)	Madake (*Phyllostachys bambusoides*)
Treatment Modification			
Stage I—Natural	KM	KyM	KyMa
Stage II—Heat Treated	KMH	KyMH	KyMaH

**Table 3:** Characteristic bands of FTIR spectra of various bamboo specimens in the frequency interval 400 to 1800 cm^−1^.

Wavenumber (cm^−1^)	Functional Group	Assignment	References
898	C-H	Bending vibration of β-glucosamine bond in cellulose	[20]
1045	C-O, C-H	Primary alcohol, guaiacyl (lignin)	[21]
1159	C-O-C	Carbohydrate	[22]
1235	CO	Guaiacyl ring with CO stretching (lignin and hemicelluloses)	[19]
1326	O-H	Phenol group (cellulose)	[19]
1460	C-H	Asymmetric bending in CH_3_ (lignin)	[23]
1505	C=C	Aromatic ring (lignin), guaiacyl elements stronger than syringyl	[19]
1600	C=C	Aromatic ring (lignin)	[19]
1737	C=O	Carbonyl groups in lignin, Stretching of acetyl or carboxylic acid (hemicelluloses)	[24,25,26,27]

## Data Availability

The data presented in this study are available on request from the corresponding author. The data are not publicly available due to private restrictions.

## Supplementary Materials

The following are available online at https://www.mdpi.com/article/10.3390/antibiotics11050569/s1, File S1: OD data—Extraction Method.
